# Novel somatic single nucleotide variants within the RNA binding protein hnRNP A1 in multiple sclerosis patients

**DOI:** 10.12688/f1000research.4436.2

**Published:** 2014-09-18

**Authors:** Sangmin Lee, Michael Levin

**Affiliations:** 1 Research Service, Veterans Affairs Medical Center, Memphis, TN, USA; 2 Department of Neurology, University of Tennessee Health Science Center, Memphis, TN, USA; 3 Department of Anatomy/Neurobiology, University of Tennessee Health Science Center, Memphis, TN, USA; 4 Neuroscience Institute, University of Tennessee Health Science Center, Memphis, TN, USA

## Abstract

Some somatic single nucleotide variants (SNVs) are thought to be pathogenic, leading to neurological disease. We hypothesized that heterogeneous nuclear ribonuclear protein A1 (hnRNP A1), an autoantigen associated with multiple sclerosis (MS) would contain SNVs. MS patients develop antibodies to hnRNP A1 ^293-304^, an epitope within the M9 domain (AA ^268-305^) of hnRNP A1. M9 is hnRNP A1’s nucleocytoplasmic transport domain, which binds transportin-1 (TPNO-1) and allows for hnRNP A1’s transport into and out of the nucleus. Genomic DNA sequencing of M9 revealed nine novel SNVs that resulted in an amino acid substitution in MS patients that were not present in controls. SNVs occurred within the TPNO-1 binding domain (hnRNP A1 ^268-289^) and the MS IgG epitope (hnRNP A1 ^293-304^), within M9.  In contrast to the nuclear localization of wild type (WT) hnRNP A1, mutant hnRNP A1 mis-localized to the cytoplasm, co-localized with stress granules and caused cellular apoptosis. Whilst WT hnRNP A1 bound TPNO-1, mutant hnRNP A1 showed reduced TPNO-1 binding. These data suggest SNVs in hnRNP A1 might contribute to pathogenesis of MS.

## Introduction

Multiple sclerosis (MS) is the most common autoimmune disease of the central nervous system (CNS) in humans, whose pathogenesis remains unknown. A number of genetic and immune studies indicate dysregulated immune responses as contributors to the pathogenesis of MS ^1– 7^. Genetic analyses show an association of MS with major histocompatibility complex (MHC) Class II human leukocyte antigen (HLA)-DRB-1 and single nucleotide polymorphisms (SNPs) related to immune function ^1, 2, 8^. Both Th1/Th17 CD4 ^+^ T-lymphocytes and immunoglobulins appear to have a causative role ^1, 2, 9^. Immunoglobulin G (IgG) responses to myelin and non-myelin targets have differentiated some MS patients from healthy controls ^9– 11^. Non-myelin antigens that are targets for immunoglobulins isolated from MS patients include neurofilaments, axonal neurofascin and RNA binding proteins, including heterogeneous nuclear ribonucleoprotein A1 (hnRNP A1) ^9, 12– 16^.

Recently, mutations in RNA binding proteins have been shown to cause neurological disease ^17– 21^. For example, a mutation (p.D263V) in the prion-like domain (PrLD) of hnRNP A1 has been shown to cause familial amyotrophic lateral sclerosis (ALS) ^22^. In addition to inherited mutations, somatic variants have also been shown to cause neurological disease ^23^. hnRNP A1 performs a number of critical cellular functions related to transcription, nucleocytoplasmic transport of mRNA and translation ^24, 25^. In addition to the PrLD, other important functional domains in hnRNP A1 include two RNA binding domains (RBDs) and M9, its nucleocytoplasmic shuttling domain ^22^. M9 binds its nuclear receptor, transportin-1 (TPNO-1, also known as karyopherin β2) and the hnRNP A1:TPNO-1 complex is transported into and out of the nucleus ^3, 9, 16, 26, 27^.

Our lab has performed extensive studies on the role of autoimmunity to hnRNP A1 in MS and human T-lymphotropic virus type 1 (HTLV-1) associated myelopathy/tropical spastic paraparesis (HAM/TSP), a viral-induced model and clinical mimic of MS ^3, 28– 30^. Initially, we discovered that HAM/TSP patients develop antibodies to hnRNP A1 that cross-react with HTLV-1-tax, indicative of molecular mimicry ^29, 31^. Next, the epitope of the HAM/TSP IgG response (AA ^293-304^) was localized to M9 (AA ^268-305^) ^32^. M9 is a bipartite phenylalanine-tyrosine nuclear localization sequence (PY-NLS) that requires binding to TPNO-1 for hnRNP A1 to shuttle between the nucleus and cytoplasm ^16, 31^ Because of the similarities between MS and HAM/TSP, we hypothesized that MS patients would also develop antibodies to hnRNP A1. In fact, antibodies isolated from MS patients, in contrast to healthy controls and Alzheimer’s patients, were also found to immunoreact with the identical hnRNP-A1-M9 epitope (AA ^293-304^) ^16^. Subsequent studies indicated that the IgG was biologically active and potentially pathogenic. For example, mono-specific antibodies to hnRNP A1 isolated from patients caused decreased neuronal firing using neuronal patch clamp in rat brain sections ^31, 33^. Further, neurons exposed to anti-hnRNP A1-M9 ^293-304^ specific antibodies resulted in neurodegeneration and neuronal death ^16, 34^. The anti-hnRNP A1-M9 ^293-304^ specific antibodies also caused changes in neuronal RNA expression that correlate with the clinical phenotype of MS and HAM/TSP patients (ie. spastic paraparesis), which was subsequently confirmed in neurons isolated from the brains of MS patients ^16^. Additional studies showed that anti-hnRNP A1-M9 ^293-304^ specific antibodies entered neurons via clathrin-mediated endocytosis and caused apoptosis in a neuronal cell line ^34^. Anti-hnRNP A1-M9 ^293-304^ specific antibodies also caused a redistribution of hnRNP A1 in neurons from nuclear to an equal distribution of nuclear and cytoplasmic localization, suggesting the antibodies interfered with M9, which is required for hnRNP A1s nuclear import ^34^. Considering: (1) the role of hnRNP A1 in cellular function; (2) variant forms of hnRNP A1 cause neurodegenerative disease, and (3) hnRNP A1 is an autoimmune target in MS patients, we hypothesized that MS patients would contain novel genomic DNA single nucleotide variants (SNVs) in hnRNP A1-M9, which when expressed, would alter cellular function and contribute to cell death.

## Methods

### Patients

All blood samples were collected according to the approved Institutional Review Board protocols (Veterans Affairs Medical Center - Memphis, Study #317164, University of Tennessee Health Science Center, Study #98-06618-FB) with patient consent. The diagnosis of MS was made using published criteria ( Supplement 1, see Results) ^35^.

### Preparation of human peripheral blood monocytes (PBMCs) and isolation of genomic DNA

Human PBMCs were isolated from fresh blood by Ficoll-Paque gradient centrifugation and washed with PBS. Genomic DNA was isolated from PBMCs using the QIAmp blood kit (Quiagen Inc., Chatsworth, CA, U.S.A.) according to manufacturer's protocol. All DNA samples were quantified using Nanodrop (Quawell) and restriction enzyme digestion methods.

### PCR primers

Specific oligonucleotides were designed from the published genomic DNA sequence of the human hnRNP A1 gene. The upstream primer 5′- CAGATAAAGGC CCTCTTTCCC -3′ (3080–3100) and the downstream primer 5′- CTCAGCTACATTAGGGTTATTGGG -3′ (3667–3690) flank a 611 bp region of the human hnRNP A1 genomic DNA containing exon 8 and exon 9.

### PCR amplification and subcloning

One microgram of genomic DNA was amplified in a reaction mixture containing the primers and KOD Hot Start DNA polymerase (Novagen). Use of this DNA polymerase has a mutation frequency of 0.10% ^36^. Before adding enzyme, the reaction mixture was heated at 95°C for 2 minutes. Amplification was carried out for 35 cycles of denaturation at 95°C for 20 s, annealing at 57°C for 10 s, and extension at 72°C for 15 s, followed by terminal elongation at 70°C for 20 s. The resulting PCR product was cloned into the pCR2.1-TOPO blunt vector (Invitrogen), yielding pCR2.1-TOPO-Blunt-hnRNP A1-611 bp, and *E. coli* TOP10 was transformed with this plasmid. Purified plasmid was digested with *EcoRI* yielding either one band (no insert) or two bands, 3.9 kbp (plasmid) and 611 bp (insert). Digests were subjected to electrophoresis on 1.5% agarose gel and visualized with the Gel Logic 200 Imaging System (Kodak). Clones that contained the 611 bp insert were sequenced ( Supplement 3).

### DNA sequencing and sequence analysis

Tue pCR2.1-TOPO-Blunt-hnRNP A1 611 bp clones were subjected directly to automated DNA sequencing (ABI 3130 X L) at the University of Tennessee Health Sciences Center Molecular Resource Center. Electropherograms were obtained and sequence quality was analyzed by Sequence Analysis Software (ABI). Sequence alignment was carried out by Nucleotide BLAST (National Center for Biotechnology Information Called genomic DNA sequences were compared to mutations (SNVs, SNPs) listed in four different public databases: (1) Exome variant server (ESV): http://evs.gs.washington.edu/EVS/, (2) Catalogue of somatic mutations in cancer (COSMIC): http://cancer.sanger.ac.uk/cancergenome/projects/cosmic/, (3) 1000 genomes; a deep catalog of human genetic variation: http://www.1000genomes.org, (4) NCBI dbSNP: ( http://www.ncbi.nlm.nih.gov/snp/).

### Cloning and expression of hnRNP-A1

cDNA encoding the entire sequence of hnRNP A1 (WT) was cloned into the expression vector pTriEx™5 Ek/LIC vector (Novagen) and transfected into SK-N-SH cells, a neuroblastoma cell line (ATCC - American Type Culture Collection). The amplified open reading frame (ORF) of hnRNP A1 was subcloned into *Bam* HI and *Hin*d III sites of modified pGEX-6p-1 vector to create recombinant *E. coli* expression vectors for gluthathione S-transferase (GST) full down assay.

### Primers and site-directed mutagenesis

The primers for mutagenesis by PCR were designed basically according to the manufacturer (QuikChange™ II XL Site-Directed Mutagenesis kit; Agilent Technologies, CA). Briefly, each pair of primers contained a primer-primer complementary (overlapping) sequence at the 3′- and 5′-terminus. The designed primers were used for mutagenesis of the target residues F273L, M276L and F281L in hnRNP A1. The primers for each of the variants were: (1) p.F273L - forward: CAG TCT TCA AAT **C**TT GGA CCC ATG AAG GGA GG, reverse: CCT CCC TTC A **G**G GGT CCA AAA TTT GAA GAC TG; (2) p.M276L - forward: CAG TCT TCA AAT TTT GGA CCC **C**TG AAG GGA G, reverse: CCT CCC TTC ATG GGT CCA A **G**A TTT GAA GAC TG; (3) p.F281L - forward: C ATG AAG GGA GGA AAT **C**TT GGA GGC AGA AGC TC, reverse: GA GCT TCT GCC TCC AA **G** ATT TCC TCC CTT CAT G. All variant sites were located in hnRNPA1-M9 and both forward and reverse primers shared the region in question. The melting temperature ( *T*_m_) was calculated using the formula provided by the manufacturer Agilent Technologies: *T*_m_ = *81.5+0.41(%GC)-675/N-% mismatch.* Here, *N* is the primer length in bases. All the primers were synthesized by Genelink (Hawthorne, NY). Mutagenic reaction was performed in 50 µl of PCR mix containing 10 ng of pTriEx-5 Ek/LIC-hnRNP A1(WT) or pGEX-6p-1-hnRNP A1(WT) as template, 200 nM primer and 2.5 U Pfu DNA polymerase. The PCR temperature profile was: an initial denaturation at 95°C for 1min, followed by 18 cycles with each at 95°C for 50 sec, 60°C for 50 sec and 68°C for 1 kb/min, and a final extension at 68°C for 7 min. The PCR products of Site-Directed Mutagenesis were transformed into *E. coli* XL10-Gold competent cells and isolated using Qiagen miniprep kits (Qiagen, Germany).

### Transfection

DNA complexes prepared using a DNA (μg) to Lipofectamine ^®^ 2000 (μl) ratio of 1:2.5 for SK-N-SH cell line. For hnRNP A1 relocalization experiments, the human hnRNP A1 (WT or variant) cDNA was transfected into SK-N-SH cells (70–80% confluence) using Lipofectamine 2000 (Invitrogen, Carlsbad, CA) according to the manufacturer's instructions. After 5 hours incubation, the transfection mixture was removed from each well and replaced with DMEM containing 10% FBS. Fresh medium was conditioned for 24 h before relocalization analysis of hnRNP A1 by immunocytochemistry.

### Immunocytochemistry

SK-N-SH Cells (ATCC HTB-11) were grown on poly- l-lysine-coated cover slips and were transfected using Lipofectamine 2000. Cells were then rinsed with PBS, fixed with 4% paraformaldehyde, permeabilized with cold acetone, and blocked in PBS containing 5% BSA. Primary antibodies used were: rabbit anti-TDP-43 (1:1000, Millipore, catalog #ABN271), rabbit anti-active caspase-3 (1:50, Millipore, catalog #AB3623), rabbit anti-Neuron specific beta III tubulin (NTB3) (1:1000, Abcam, catalog #ab18207) and biotinylated mouse anti-strep-Tag II (1:1000, GenScript, catalog #A01737). Secondary antibodies were: Texas Red conjugated goat anti-rabbit IgG (1:300, Vector, catalog #TI-5000 and FITC conjugated strepavidin (1:300, Vector, catalog #SA-5001). Primary antibodies were diluted in blocking solution incubated with each coverslip for overnight at 4°C. Cells were then washed with PBS and incubated in secondary antibody for 1 hr. Cells were then washed with PBS and mounted in Prolong-Gold anti-fade reagent with DAPI (Invitrogen).

### GST pull-down assay

SK-N-SH cells were cultured in Dulbecco’s Modified Eagle’s medium (BD Biosciences) supplemented with 10% fetal bovine serum, 100 U/mL penicillin G and 100 μg/mL streptomycin, at 37°C under 5% CO _2_. Cells were harvested and lysed with CytoBuster™ Protein Extraction Reagent (Millipore), containing inhibitor cocktail, homogenized for a few seconds with a handheld homogenizer and spun at 16,000 × g for 5 minutes. Supernatants were used for GST-pull down assays. Glutathione-Sepharose 4B beads coupled with GST-hnRNP A1 (WT or variant), which includes the Transportin 1-binding domain, were incubated for 1 h at 4°C with 600 μL of the cell lysates in CytoBuster™ Protein Extraction Reagent and protease inhibitors. After washing the beads three times with 600 μL of 10 mM PBS (10 mM Na _2_HPO _4_, 140 mM NaCl, 2.7 mM KCl, 1.8 mM KH _2_PO _4_, pH 7.4) and protease inhibitors, proteins bound to the beads were analyzed by 8–16% SDS-PAGE followed by immunoblotting with rabbit polyclonal GST antibody (1:1000, Millipore, catalog #06-332), mouse monoclonal Transportin 1 antibody (1:1000, Millipore, catalog #05-1515) and mouse monoclonal TDP-43 antibody(1:1000, Millipore, catalog #MABN45). The immunoreactive bands were visualized using enhanced chemiluminescence.

## Results

### Novel somatic DNA SNVs are contained within the TPNO-1 binding domain and MS IgG epitope of hnRNP A1-M9 in MS patients

**Figure 1.**
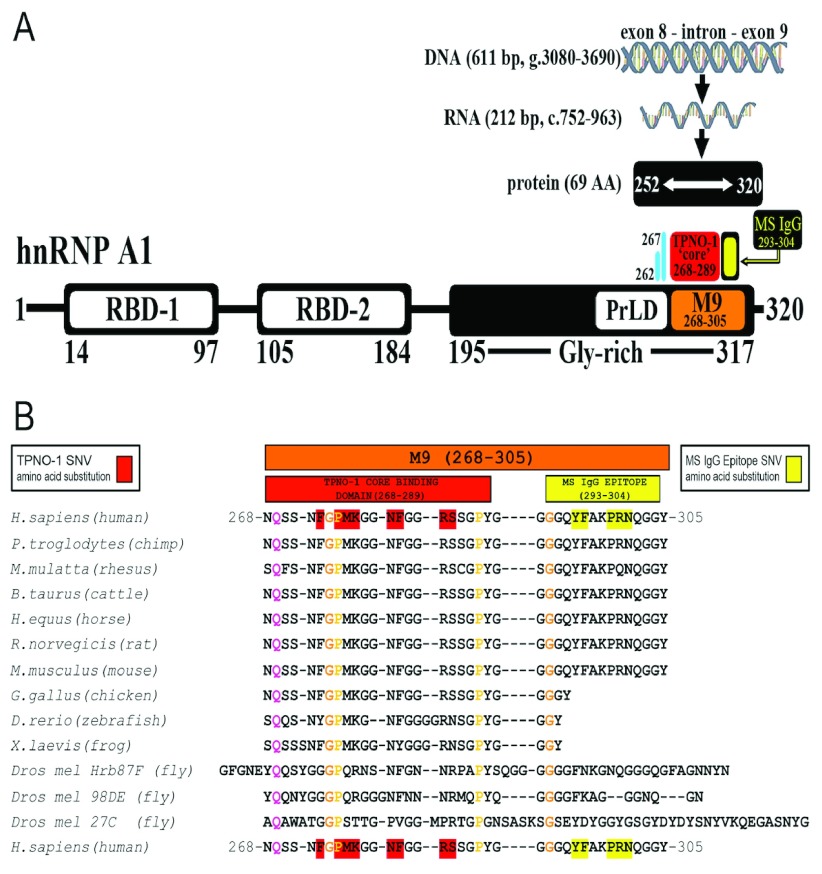
Functional domains and sequence alignment of hnRNP A1. **A**: Domain schematic of hnRNP A1. The sequence shown is isoform A (NP_002127). Isoform B contains a 52 amino acid insert following AA ^251^, resulting in a 372 amino acid protein (not shown). The RNA binding domains (RBD 1 and 2) are contained within the N-terminal half of hnRNP A1. The prion-like domain (PrLD, AA ^233-267^), M9 (AA ^268-305^) and the C-terminus (AA ^306-320^) are shown. M9 (orange) contains the ‘core’ transportin-1 binding domain (TPNO-1, AA ^268-289^, red) and the MS IgG epitope (AA ^293-304^, yellow). Some literature indicates that the PrLD and TPNO-1 binding domain may overlap, such that the PrLD region includes AA ^233-272^ and the TPNO-1 binding domain includes AA ^263-289^, with the a resulting overlap of AA ^263-272^ ( Supplement 2). The amplicon included DNA from exons 8 and 9 with the intervening intron. The expressed protein included the PrLD that contained ALS-associated mutations (p.D262V (familial), p.N267S (sporadic)(blue)), as well as M9 and the C-terminus of hnRNP A1. (bp base pair). **B**: Sequence alignment of hnRNP A1-M9. Human sequences are 100% conserved in mammals, except for *M. mulatta* (rhesus). There is also high sequence conservation between orthologs (mammals, non-mammalian vertebrates ( *G. gallus* - chicken, *D. rerio* - zebrafish, *X. laevis* - frog) and invertebrates ( *D. melanogaster* - fruit fly). SNVs resulting in amino acid substitutions in the TPNO-1 core domain are highlighted in red boxes. SNVs resulting in amino acid substitutions in the MS IgG epitope are highlighted in yellow boxes. Colored amino acids are present for clarity identifying conserved sequences through all species. Black lines (gaps) are inserted between residues so that similar or identical amino acids are aligned in each column. ( http://www.bioinformatics.org/strap/).

We sequenced a 611 bp region of hnRNP A1 genomic DNA inclusive of exons 8 and 9 with intervening introns (NP_002127.1, Chromosome 12q13.1, DNA g.3080-3690, RNA (cDNA) c.752-963, protein AA ^252-320^) ( Figure 1A) isolated from the PBMCs of patients with MS: relapsing remitting MS (RRMS, n=5), secondary progressive (SPMS, n=5) and primary progressive MS (PPMS, n=4) and healthy controls (HC, n=6) ( Supplement 1). The expressed sequence included: the C-terminal of the PrLD (AA ^252-267^), M9 (AA ^268-305^) and the residual C-terminus of hnRNP A1 (AA ^306-320^) ( Figure 1A). This region also includes the ‘core’ TPNO-1 binding domain (AA ^268-289^) and the MS IgG epitope (AA ^293-304^). Some literature indicates that the PrLD and TPNO-1 binding domain may overlap, such that the PrLD region includes AA ^233-272^ and the TPNO-1 binding domain includes AA ^263-289^, with the a resulting overlap of AA ^263-272^ ( Supplement 2) ^22, 26^. In addition, the previously reported mutations (p.D262V (familial), p.N267S (sporadic)) that cause ALS are also contained within the target sequence ( Figure 1A, Supplement 2) ^22^. A small percentage of clones from each individual contained genomic DNA SNVs, indicative of these being somatic SNVs derived from a small percentage or subset of PBMC. SNVs were compared to those found in four different databases (see Methods and Supplement 2).

Of the six HCs, zero SNVs resulted in an amino acid substitution within the TPNO-1 binding domain, MS IgG epitope or M9 from the 481 clones that were sequenced ( Table 1, Supplement 2, Supplement 3). One individual had a likely benign variant which did not result in a change in the associated amino acid (c.900A>G, p.R300R) ( Supplement 2), and three others had SNVs in the C-terminal region (c.922T>C, p.S308P; c.949G>A, p.G317S; c.952A>G, p.R318G), which altered the amino acid sequence ( Table 1, Supplement 2, Supplement 3). The SNV at AA ^308^ was previously reported and not associated with disease ( http://www.ncbi.nlm.nih.gov/snp). These data, in which SNVs in hnRNP A1 are a rare event, are consistent with the finding in sporadic (1 of 305) and familial ALS (1 of 212) patients where most did not have mutations by whole exome sequencing ^22^.

**Table 1.** hnRNP A1 single nucleotide variants that resulted in an amino acid substitution. HC: Healthy Control. RRMS: Relapsing Remitting Multiple Sclerosis. SPMS: Secondary Progressive Multiple Sclerosis. PPMS: Primary Progressive Multiple Sclerosis. TPNO-1: Transportin 1. PRLD: Prion-like Domain. AA: aminoacid. SNV: single nucleotide variant.

EXON>	EXON 8 >																									<EXON 9	<EXON
DOMAIN>	251-----------------PrLD------------------>267				268<-------------------------------------------------------------------------------M9-------------------------------------------------------------------------------------->305															306<--------------------C-terminal-------------------->320							<DOMAIN
DOMAIN>	(233)				268<-------------------------------TPNO-1 Binding Domain------------------------------>289									293<-----------------MS IgG Epitope--------------->304													<DOMAIN
AA SNV site>	252	259	263	265	273	275	276	277	280	281	284	285		295	296	299	300	301		308	313	314	317	318	319	320	<AA SNV site
**PPMS-1**				**793 A>G**		**823 C>T**																	**950 G>A**				**PPMS-1**
				**N265D**		**P275S**																	**G317D**				
**PPMS-2**								**831 G>T**																			**PPMS-2**
								**K277N**																			
**PPMS-3**				**793 A>G**							**850 A>G**																**PPMS-3**
				**N265D**							**R284G**																
**PPMS-4**					**817 T>C**					**841 T>C**		**853 A>G**						**901 A>G**		**922 T>G**							**PPMS-4**
					**F273L**					**F281L**		**S285G**						**N301D**		**S308P**							
																											
**SPMS-1**			**787 T>C**													**896 C>T**	**898 C>T**					**941 A>G**				**958 T>C**	**SPMS-1**
			**F263L**													**P299L**	**R300S**					**Y314C**				**F320L**	
**SPMS-2**																											**SPMS-2**
																									
**SPMS-3**					**817 T>G**																						**SPMS-3**
					**F273L**																						
**SPMS-4**	**755 G>A**													**884 A>G**	**886 T>C**			**902 A>G**									**SPMS-4**
	**S252N**													**Y295C**	**F296L**			**N301S**									
**SPMS-5**																											**SPMS-5**
																									
																											
**RRMS-1**																					**937 A>G**				**955 A>G**		**RRMS-1**
																					**S313G**				**R319G**		
**RRMS-2**							**826 A>C**		**839 A>G**													**940 T>C**					**RRMS-2**
							**M276L**		**N280S**													**Y314H**					
**RRMS-3**																											**RRMS-3**
																									
**RRMS-4**																											**RRMS-4**
																									
**RRMS-5**		**775 A>G**																									**RRMS-5**
		**S259G**																									
																											
**HC-1**																											**HC-1**
																									
**HC-2**																											**HC-2**
																										
**HC-3**																											**HC-3**
																									
**HC-4**																				**922 T>C**							**HC-4**
																				**S308P**							
**HC-5**																								**952 A>G**			**HC-5**
																								**R318G**			
**HC-6**																							**949 G>A**				**HC-6**
																							**G317S**				
DOMAIN>					268<-------------------------------TPNO-1 Binding Domain------------------------------>289									293<-----------------MS IgG Epitope--------------->304													<DOMAIN
DOMAIN>	233<-----------------PrLD---------------->267				268<-------------------------------------------------------------------------------M9-------------------------------------------------------------------------------------->305															306<--------------------C-terminal-------------------->320							<DOMAIN
EXON>	EXON 8 >																									<EXON 9	<EXON

Of the five RRMS patients in which 358 clones were sequenced, one patient had two novel SNVs contained within the TPNO-1 binding domain that resulted in an amino acid substitution (c.826A>C, p.M276L; c.839A>G, p.N280S) ( Table 1, Supplement 2, Supplement 3, Data availability). None of the other RRMS patients had changes within the TPNO-1 binding domain or M9. Other SNVs that resulted in an amino acid substitution included those within the C-terminal region (c.937A>G, p.S313G; c.940T>C, p.Y314H; c.955A>G, p.R319G) and one within the PrLD (c.775A>G, p.S259G). These SNVs are also novel ( Table 1, Supplement 2, Data availability). There was a single SNV (c.963A>G) that did not alter the stop codon amino acid sequence ( Supplement 2).

Of the five SPMS patients, one had a novel SNV that resulted in an amino acid substitution in the ‘core’ TPNO-1 binding domain (c.817T>G, p.F273L). Although there was a somatic SNV contained within this codon in the COSMIC database (in patients with cancer), its SNV and amino acid change were different (c.818T>G, p.F273C). A second patient had a novel SNV contained within the PrLD (c.755G>A, p.S252N), which also aligned with a somatic SNV in the COSMIC database (c.755G>T, p.S252I), but yielded different amino acids. A third patient had an SNV within the PrLD (c.787T>C, p.F263L), which also aligned with a somatic SNV contained within this codon in the COSMIC database (c.789T>G, p.F263L). In contrast to HC, RRMS or PPMS, novel SNVs that resulted in an amino acid substitution in SPMS predominated within the MS IgG binding epitope of M9 (c.884A>G, p.Y295C; c.886T>C, p.F296L; c896C>T, p.P299L; c.898C>T, p.R300S; c.902A>G, p.N301S) ( Table 1, Supplement 2, Supplement 3, Data availability). Overall, of the 355 clones that were sequenced from the five SPMS patients, 8 (2.25%) were contained within the PrLD and M9 domains of which 6 were within M9, and 5 were exclusive to the MS IgG epitope ( Table 1, Supplement 2, Supplement 3). Like HC and RRMS, there were several SNVs contained in the C-terminal region of hnRNP A1 (c.941A>G, p.Y314C; c.958T>C, p.F320L) ( Table 1, Supplement 2, Supplement 3).

In contrast to HC, all four PPMS patients, had novel somatic SNVs that resulted in an amino acid substitution and were contained within the ‘core’ TPNO-1 binding domain of hnRNP A1 as follows: patient 1 (c.823C>T, p.P275S), patient 2 (c.831G>T, p.K277N), patient 3 (c.850A>G, p.R284G), and patient 4 (c.817T>C, p.F273L; c.841T>C, p.F281L; c.853A>G, p.S285G) ( Table 1, Supplement 2, Data availability). Patients 1 and 3 each had a novel SNV within the PrLD region (c.793A>G, p.N265D). Thus, of the 317 clones that were sequenced, 2.84% were contained within the M9 or PrLD domains, two-thirds of which were exclusive to the TPNO-1 binding domain ( Supplement 3). Other SNVs were contained within the MS IgG binding epitope (c.901A>G, p.N301D) or the C-terminal region (c.922T>G, p.S308P; c.950G>A, p.G317D) ( Table 1, Supplement 2, Supplement 3, Data availability). Only the c.817T>C, p.F273L SNV aligned with a somatic SNV within the same codon in the COSMIC database (c.818T>G, p.F273C), but again, both the SNV and amino acid substitution differed.

The TPNO-1 binding domain is highly conserved within mammals and evolutionarily conserved between species ( Figure 1B). Specifically, the TPNO-1 binding domain is 100% conserved in mammals, except for five mutations contained only within the rhesus monkey ( *Macaca mulatta*) (only one of which overlapped with the SNVs we discovered (AA ^300^) ( Figure 1B)). Further, amino acid sequences were highly conserved between species, as shown by the orthologs between mammals, bird ( *Gallus gallus*), fish ( *Danio rerio*) and frog ( *Xenopus laevis*) as well as with the fruit fly ( *Drosophila melanogaster*) ( Figure 1B). Taken together, these data indicate that variants in this highly conserved domain may have pathological consequences, which might contribute to human disease.

### Disease-associated SNVs of the TPNO-1 binding domain of hnRNP A1-M9 result in mis-localization of hnRNP A1 into cytoplasmic stress granules and cellular apoptosis

**Figure 2.**
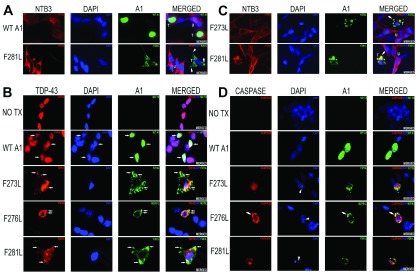
Transfection of WT and mutant forms of hnRNP A1 into SK-N-SH cells. ***A**. Localization of WT and F281L hnRNP A1*. (upper panel) hnRNP A1 localizes to the nuclei of cells transfected (T) with WT hnRNP A1. Untransfected (U) cells are also present in the same field. (Lower panel) In contrast, mutant hnRNP A1 (F281L) mis-localizes to the cytoplasm including cellular processes (arrowhead) in a granular pattern, consistent with stress granule (SGs) formation (arrows) (NTB3-Neuron specific beta III tubulin). ***B**. Co-localization of hnRNP A1 and TDP-43*. TDP-43 localizes to nuclei of neurons (‘no tx’) (pink or red signal). WT hnRNP A1 co-localizes with TDP-43 in nuclei (arrows). In contrast, mutant forms of hnRNP A1 (F273L, M276L, F281L) predominantly mis-localize to the cytoplasm and co-localize with TDP-43 positive SGs (arrows, yellow). Some TDP-43 remains in the nucleus (pink or red signal). Figures 2C and D: Apoptosis caused by mutant hnRNP A1 in SK-N-SH neurons. **C**. Transfection of mutant hnRNP A1 (F273L, F281) result in apoptotic blebs in transfected cells (T) (arrows), in contrast to untransfected (U) cells in the same field under identical conditions. **D**. Transfection of WT hnRNP A1 localized to the nucleus and showed no evidence of active caspase-3 labeling. In contrast, mutant hnRNP A1 (F273L, M276L, F281L) predominantly localized to the cytoplasm of cells that stained with active caspase-3, many of which with fragmented nuclei (arrowheads), both of which are indicative of apoptosis. There was also co-localization of active caspase-3 and mutant hnRNP A1 (arrow).

**Figure 3.**
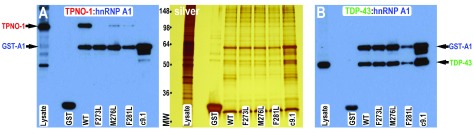
Western blots of hnRNP A1 binding with TPNO-1 and TDP-43. GST-tagged WT or mutant forms of hnRNP A1 were bound to Glutathione Sepharose 4B beads, protein lysates of SK-N-SH cells were applied to columns, and the resulting eluent was probed for either TPNO-1 ( **A**) or TDP-43 ( **B**). **A**. There was strong binding between WT hnRNP A1 and TPNO-1. In contrast, there was no binding between mutant hnRNP A1 (F273L) and TPNO-1, and markedly reduced binding between mutant hnRNP A1s (M276L, F281L) and TPNO-1. **B**. There was no change in binding between WT and mutant forms of hnRNP A1 and TDP-43.

A total of nine novel SNVs that resulted in an amino acid substitution were discovered in MS patients within the ‘core’ TPNO-1 binding domain of hnRNP A1-M9. hnRNP A1 has a number of functions, including the transport of nascent mRNA from the nucleus to the cytoplasm. hnRNP A1 shuttles between the nucleus and cytoplasm and binds TPNO-1, which is required for its nuclear import ^16, 26, 27^. At equilibrium, hnRNP A1 is predominantly found in the nucleus ^37, 38^. Considering the disease-associated SNVs are contained within a highly conserved region of hnRNP A1 that plays a critical role in cellular function, we hypothesized that altered hnRNP A1 would change hnRNP A1 localization, TPNO-1 binding and induce cellular damage. To test this hypothesis, we performed a number of experiments. First, we manufactured three different hnRNP A1 mutations (by site-directed mutagenesis) contained within its TPNO-1 binding domain (F273L, M276L and F281L), transfected each mutant into SK-N-SH cells and examined the cells for hnRNP A1 localization relative to transfection of Wild Type (WT) hnRNP A1. As shown in Figure 2A (upper panel), WT hnRNP A1 almost completely localized to the nucleus of SK-N-SH cells. In contrast, mutant forms of hnRNP A1 localized to the cytoplasm of cells ( Figure 2A, lower panel). Localization within the cytoplasm was not diffuse, but granular, suggestive of stress granule (SG) formation ( Figure 2A, lower panel, arrows). There was also localization within cellular processes ( Figure 2A, lower panel, arrowhead). To confirm that mutant hnRNP A1 was present in SGs, we double-labeled SK-N-SH cells that contained the transfected mutant hnRNP A1 with anti-TDP-43 antibodies. As shown in Figure 2B, like hnRNP A1, TDP-43 was localized to nuclei (without transfection). In addition, WT hnRNP A1 and TDP-43 co-localized within the nuclei of SK-N-SH cells. In contrast, mutant hnRNP A1 (F273L, M276L and F281L) co-localized with TDP-43 within the cytoplasm of cells ( Figure 2B). Considering recent data indicating binding between hnRNP A1 and TDP-43; co-localization of mutant hnRNP A1 (p.D262V) to TDP-43 containing SGs; the role of TDP-43 in SG formation, and the localization of hnRNP A1 in SGs in stress activated cells, these experiments confirm that mutant hnRNP A1 is contained within TDP-43 positive SGs ^22, 39– 42^. Because TPNO-1 is required for hnRNP A1 nucleocytoplasmic transport, we hypothesized that mutant hnRNP A1 would alter its binding to TPNO-1. In these experiments, protein lysates purified from SK-N-SH cells were incubated with either WT or mutant GST-tagged-hnRNP A1 bound to Glutathione-Sepharose 4B beads. The resultant eluent was then probed for TPNO-1. As shown in Figure 3A, western blots showed immunoreactivity for TPNO-1 protein with WT-hnRNP A1, indicative of TPNO-1’s binding to hnRNP A1. In contrast, there was significantly reduced binding between mutant forms of hnRNP A1 and TPNO-1 ( Figure 3A). These experiments show that mutations in the TPNO-1 binding domain of hnRNP A1-M9 alter TPNO-1 binding to hnRNP A1. To confirm protein binding between hnRNP A1 and TDP-43, which were visualized by immunocytochemistry ( Figure 2B), we probed the identical eluents with an anti-TDP-43 antibody. Both WT and mutant hnRNP A1 bound TDP-43 ( Figure 3B), indicative of their interaction in both the nuclei and cytoplasmic SGs in the cell line. This is consistent with other reports indicative of an interaction between hnRNP A1 and TDP-43 ^22^.

In the *in-vitro* experiments, SG formation in SK-N-SH cells formed within several hours of transfection. When we waited overnight (approximately 24 hours) the cells containing mutant hnRNP A1 developed apoptotic blebs, which contained hnRNP A1 ( Figure 2C, arrows). Apoptosis was confirmed by active caspase-3 staining ( Figure 2D). As shown in Figure 2D, SK-N-SH cells transfected with mutant hnRNP A1 showed a cytoplasmic hnRNP A1 distribution, stained positive for active caspase-3 and contained fragment nuclei, confirming apoptosis.

In summary, in contrast to WT hnRNP A1, mutant hnRNP A1 showed markedly reduced binding to its co-receptor TPNO-1, co-localized with TDP-43 within cytoplasmic SGs of cells and caused apoptosis, indicative of the potential pathogenic nature of these disease-associated SNVs in MS patients.

## Discussion

Recent studies indicate that in addition to cancer, somatic variants can cause neurological disease ^23^. In this study, we discovered novel somatic genomic DNA SNVs in MS patients. Nine were contained within the ‘core’ TPNO-1 binding domain of hnRNP A1-M9 (AA ^268-289^). Three additional SNVs (c.793A>G, p.N265D (in two patients); c.787T>C, p.F263L) included amino acids within the PrLD - M9 overlap region (AA ^263-267^), which also bind TPNO-1 ^45^. These variants were in a region of hnRNP A1 that are adjacent to mutations shown to cause ALS (p.D262V, p.N267A). Interestingly, 8 of these 12 SNV’s that involved hnRNP A1-M9 binding to TPNO-1 occurred in PPMS patients. In addition, two hnRNP A1 SNVs were contained exclusively within the PrLD (c.755G>A, p.S252N; c.775A>G, p.S259G). There were also six novel SNVs that resulted in an amino acid substitution within the MS IgG epitope of M9 (AA ^293-304^), five of which segregated to patients with SPMS. Finally, there were nine SNV’s in the C-terminal of hnRNP A1 (AA ^306-320^), occurring with similar frequency in HCs and MS patients. The overall somatic SNV rate (based on the number of clones sequenced) for the M9 target sequence was: PPMS - 2.21%, SPMS - 1.69%, RRMS - 0.56%, HC - 0%. If one includes the PrLD (a domain shown to be critical to hnRNP A1 function), the rates increase in PPMS, SPMS and RRMS to 2.84%, 2.25% and 0.84% respectively. None were identical to somatic mutations in the COSMIC database (n = 981,720 samples, n = 1,292,597 unique variants). We utilized a PCR - cloning technique that has been fine-tuned for more than a decade and shows a mutation rate of approximately 0.1% in more than 46,000 clones that were examined ^36^. The rates in progressive MS patients exceed this error rate by more than a log. In addition, under identical conditions, there were no mutations in the M9 target sequence or the PrLD domain in the HCs we examined. Thus, these results are unlikely to be due to PCR errors. Importantly, there was little or no overlap with either SNVs or SNPs reported in four different databases.

hnRNP A1 was one of the first RNA binding proteins shown to shuttle into and out of the nucleus ^37, 38^. Nucleocytoplasmic transport is dependent upon binding between the M9 domain (AA ^268-305^) of hnRNP A1 to TPNO-1, in order for this complex to pass through the nuclear pore. M9 acts as both an NES and NLS. M9 is a bipartite PY-NLS whose three-dimensional structure and binding contacts with TPNO-1 are well characterized ^26, 27, 43^. Specifically, M9 contains three binding epitopes ( Table 2): a hydrophobic (273-FGPM-276) domain, a basic residue (522R) and a C-terminal RX _2-5_ PY motif, each connected by ‘linker’ residues ^26, 27, 43^. Each epitope, as well as individual amino acids within an epitope, conveys varying degrees of binding activity to TPNO-1 ^26, 43, 44^. For example, mutant P275A dramatically inhibits nucleocytoplasmic transport and substitution of 273-FGPM-276 with 273-AAAA-276, completely abolishes TPNO-1 binding and nucleocytoplasmic transport ^37, 43, 44^. Our data closely align with these findings. For instance, genomic SNVs in MS patients occurred at F273L, F275S, and M276L - all contained within epitope 1. Experimentally, we showed that transfection of F273L and M276L mutants caused mis-localization of hnRNP A1, SG formation (co-localizing with TDP-43), cellular apoptosis and diminution of TPNO-1 binding. Mutant F281L caused similar results. Interestingly, F273, F281 and R284 all have two or more side chains that bind TPNO-1, thus are critical contact points between M9 and TPNO-1 ^26^. MS patients also had an SNV at R284G. Although we did not test this variant, a parallel substitution in the RNA binding protein fused in sarcoma (FUS) (R522G) ( Table 2) caused a five-fold decrease in TPNO-1 binding and cytoplasmic mis-localization of FUS ^27^. Like hnRNP A1, FUS is an hnRNP, which at equilibrium localizes to the nucleus and contains a bipartite PY-NLS that binds TPNO-1 ^27^. Interestingly, mutations in FUS have been shown to cause ALS and frontotemporal lobe dementia ^17^.

**Table 2.** Binding epitopes of the PY-NLS’s of hnRNP A1 and fused in sarcoma (FUS). Aligned PY-NLS sequences of hnRNP A1-M9 and FUS-NLS are shown. The three binding epitopes of the PY-NLS are shaded yellow (epitope 1 - hydrophobic), blue (epitope 2 - basic) and red (epitope 3 - C-terminal) ^27, 43^. SNVs resulting in an amino acid substitution (hnRNP A1) and missense mutations (FUS) are shown by gray shading of the amino acid sequence number. Amino acid SNVs associated with multiple sclerosis (MS) in hnRNP A1 are shown above the primary sequence and mutations associated with amyotrophic lateral sclerosis (ALS) in FUS are shown below the primary sequence. Two SNVs (*) in hnRNP A1-M9 align with mutations in the FUS NLS: hnRNP A1-M9 AA ^275^ with FUS NLS AA ^510^ and hnRNP A1-M9 AA ^284^ with FUS NLS AA ^522^. Stop codons, frame shift mutations, insertions and deletions not included. Notes: ^1^R514G/S, ^2^R514S, G515C, ^3^R514S, E516V ^17^.

														*												*					
hnRNP A1 AA sequence #	262	263	264	265	266	267	268	269	270	271	272	273	274	275	276	277	278	279	280	281	282	283	(-)	(-)	(-)	284	285	286	287	288	289
hnRNP A1 AA SNV (MS)		L		D								L		S	L	N			S	L						G	G				
hnRNP A1 AA sequence	D	F	G	N	Y	N	N	Q	S	S	N	F	G	P	M	K	G	G	N	F	G	G	(-)	(-)	(-)	R	S	S	G	P	Y
FUS AA sequence											G	P	G	K	M	D	S	R	G	E	H	R	Q	D	R	R	(-)	E	R	P	Y
FUS AA mutation (ALS)											D			E/R			P	G/S ^1,2,3^	C ^2^	V ^3^	P/Q	G/K			G/H/L	G			S/T/W	L	
FUS AA sequence #											507	508	509	510	511	512	513	514	515	516	517	518	519	520	521	522	(-)	523	524	525	526

Importantly, none of the SNVs contained within hnRNP A1 - M9 or the PrLD has been reported previously. For decades, the only certain genetic risk factor for MS was with MHC Class II HLA-DRB1 ^8^. Genome Wide Association Studies (GWAS) have uncovered novel genetic associations with MS ^1, 2, 45^ including with the interleukin-2 receptor-α and interleukin-7 receptor genes ^45^. Subsequent studies using several thousand MS cases and controls, which analyzed hundreds of thousands of autosomal SNPs, confirmed the association of MS with major MHC Class II HLA-DRB1 (DRB1 *15:01, *15:03, *13:03) and the protective effect MHC Class I HLA-A*02 ^1, 8^. Additional studies showed a total of 48 new and 49 known non-MHC SNPs associated with MS ^2^. Interestingly, the functions of the vast majority of the SNP’s were related to CD4 ^+^ T-lymphocyte and immune regulation ^1, 8^. This is important, considering the role that T-lymphocytes and the immune response play in the pathogenesis of MS. A few were potentially associated with neurodegeneration ^46^. Further, >95% of the SNPs were intronic or intergenic, with only a few SNPs involving exons, in contrast to the somatic SNVs discovered here ^1, 2^. In addition to GWAS, whole exome sequencing (WES) is being used to examine differential gene expression in MS patients. In contrast to GWAS, which detects known SNPs and utilizes statistical analyses designed to reveal common variants, WES is designed to discover novel, rare pathologic variants ^8^. One of the genes identified by GWAS was CYP27B1, which encodes an enzyme of the same name that converts 25-hydroxyvitamin D to 1,25 hydroxyvitamin D, the biologically active form of vitamin D ^1, 8^. A single individual from 43 MS families was found to have a rare p.R389H genetic variant in CYP27B1, which resulted in complete loss of enzyme activity ^8, 47, 48^. However, unaffected relatives of the individual also carried the identical variant, which has a high frequency in the general population ^48^.

Thus, it is clear that an individual’s genetic background makes an important contribution to the pathogenesis of MS. This supports the tripartite hypothesis that an environmental trigger in a genetically susceptible individual causes an autoimmune response to CNS antigens that result in the pathology observed in the brain and spinal cord of MS patients. Potential environmental triggers include viral infection, low vitamin D levels and sun exposure ^49– 51^. During the relapsing phase of MS, Th1 and Th17 CD4 ^+^ T-lymphocyte responses appear to predominate and correlate with focal MS plaque formation in the CNS ^5, 52, 53^. As MS evolves into a secondary progressive phase, CNS damage becomes more diffuse ^5, 6, 53^. Immune cells also become more diffuse and IgG containing plasma cells, B-lymphocytes and macrophage/microglia response predominate ^7, 53^. Many of these latter features are also characteristic of primary progressive MS ^7^. MS is also characterized by increased oxidative stress (in PBMC and brain), which can cause DNA damage and somatic mutations ^54– 56^. Further, T-lymphocytes isolated from MS patients that contain somatic mutations have been shown to react to myelin peptides ^57^. Specifically, T-cell clones mutant for the hypoxanthine guanine phosphoribosyltransferase (HPRT) gene were found only in MS patients and not in HCs ^57, 58^. The MS patients studied had ‘chronic progressive MS’, clinically similar to the PPMS patients in this study ^57^. The T-cell clones recognized and proliferated to myelin basic protein peptides ^57^. These data suggest that T-cells that undergo clonal expansion are more prone to somatic mutations and that clones containing somatic mutations contribute to the pathogenesis of MS ^57, 58^. How might variants in hnRNP A1 contribute to the pathogenesis of MS? Although direct evidence is not yet available and requires further study, the molecular consequences of abnormal forms of hnRNP A1 on cellular function may have profound effects on the immune system. For example, as it relates to the environment, hnRNP A1 regulates the synthesis of several viruses including human immunodeficiency virus, HTLV-1, and human rhinovirus ^59– 61^. Immunologically, hnRNP A1’s nucleocytoplasmic shuttling and RNA binding specificity is required for myelopoiesis and modulation of immune-mediated programmed cell death ^62– 64^. Further, apoptotic blebs (which we showed in this study to contain hnRNP A1) have profound effects on the immune response, as they are believed to initiate and perpetuate autoimmune diseases such as systemic lupus erythematosus ^65, 66^.

In addition, genetic mutations in hnRNP A1 alter neuronal function. For example, an inherited mutation in the PrLD of hnRNP A1 resulted in its mis-localization to TDP-43 positive cytoplasmic SGs and thus is thought to cause familial ALS ^22^. A second mutation in the PrLD was also discovered in a sporadic case of ALS ^22^. Interestingly, in addition to inherited and germ line mutations (including SNPs), recent data indicate that acquired mutations cause neurological disease ^23^. For example, both familial (inherited) and acquired (‘ *de novo*’, somatic variants) of doublecortin cause subcortical band heterotopia (SBH), a neuronal migration syndrome that results in epilepsy and intellectual disability ^67^. In contrast to inherited mutations, somatic mutations may only be present in specific cell lineages ^23^. In SBH, somatic mutations are only found in the DNA of neurons and lymphocytes, and not in other tissues (a ‘mosaic’) ^23, 67^. To date, we have only discovered somatic mutations in PBMCs of MS patients. Future studies will address their presence in CNS tissues of MS patients. If present in the CNS, abnormal forms of hnRNP A1, which have profound effects on neurons, might also damage astrocytes and oligodendroglia, and could contribute to neurodegeneration present in MS, particularly in PPMS where the majority of the SNVs were located.

In summary, we discovered novel SNVs in MS patients. The SNVs involve the M9 nucleocytoplasmic binding domain of hnRNP A1, which when transfected into a cell line, resulted mis-localization of hnRNP A1 to cytoplasmic stress granules and cellular apoptosis. Future studies are required to replicate this data, expand it to include a broader spectrum of genes, a greater number of MS patients and patients with other chronic inflammatory diseases such as rheumatoid arthritis. In addition, we also plan to perform functional studies of the SNVs in immune and nervous system cells of MS patients.

## Data availability

Single nucleotide variants (SNVs) (somatic) in MS patients submitted to dbSNP ^68^

**Table:** 

#	HGVS NAME	LOCAL ID	ss
1	NM_002136.2:c.755G>A	NP_002127.1:c.755G>A	ss1056389899
2	NM_002136.2:c.775A>G	NP_002127.1:c.775A>G	ss1056389900
3	NM_002136.2:c.787T>C	NP_002127.1:c.787T>C	ss1056389901
4	NM_002136.2:c.793A>G	NP_002127.1:c.793A>G	ss1056389902
5	NM_002136.2:c.826A>C	NP_002127.1:c.826A>C	ss1056389903
6	NM_002136.2:c.839A>G	NP_002127.1:c.839A>G	ss1056389904
7	NM_002136.2:c.937A>G	NP_002127.1:c.937A>G	ss1056389905
8	NM_002136.2:c.940T>C	NP_002127.1:c.940T>C	ss1056389906
9	NM_002136.2:c.955A>G	NP_002127.1:c.955A>G	ss1056389907
10	NM_002136.2:c.817T>G	NP_002127.1:c.817T>G	ss1056389908
11	NM_002136.2:c.817T>C	NP_002127.1:c.817T>C	ss1056389909
12	NM_002136.2:c.823C>T	NP_002127.1:c.823C>T	ss1056389910
13	NM_002136.2:c.831G>T	NP_002127.1:c.831G>T	ss1056389911
14	NM_002136.2:c.841T>C	NP_002127.1:c.841T>C	ss1056389912
15	NM_002136.2:c.850A>G	NP_002127.1:c.850A>G	ss1056389913
16	NM_002136.2:c.853A>G	NP_002127.1:c.853A>G	ss1056389914
17	NM_002136.2:c.884A>G	NP_002127.1:c.884A>G	ss1056389915
18	NM_002136.2:c.886T>C	NP_002127.1:c.886T>C	ss1056389916
19	NM_002136.2:c.896C>T	NP_002127.1:c.896C>T	ss1056389917
20	NM_002136.2:c.898C>T	NP_002127.1:c.898C>T	ss1056389918
21	NM_002136.2:c.902A>G	NP_002127.1:c.902A>G	ss1056389919
22	NM_002136.2:c.901A>G	NP_002127.1:c.901A>G	ss1056389920
23	NM_002136.2:c.922T>G	NP_002127.1:c.922T>G	ss1056389921
24	NM_002136.2:c.941A>G	NP_002127.1:c.941A>G	ss1056389922
25	NM_002136.2:c.950G>A	NP_002127.1:c.950G>A	ss1056389923
26	NM_002136.2:c.958T>C	NP_002127.1:c.958T>C	ss1056389924

## Supplementary materials

Supplement 1: Patient demographics (xlsx)

Supplement 2: Single nucleotide variants in the target sequence of hnRNP A1 (xlsx)

Supplement 3: Number of clones containing single nucleotide variants (xlsx)
