# Supplementary material for: Novel somatic single nucleotide variants within the RNA binding protein hnRNP A1 in multiple sclerosis patients
Source: F1000Res. 2014 Sep 18;3:132. Originally published 2014 Jun 20. [Version 2] doi: 10.12688/f1000research.4436.2 (PMC4168748; doi:10.12688/f1000research.4436.2)
Supplement: Peer review history

# Peer review: Referee response for version 1

**DOI:** 10.5256/f1000research.4747.r5807

**Published:** 2014-09-10

**Authors:** Elliot M. Frohman

**Affiliations:** 1 Department of Neurology, University of Texas Southwestern Medical Center at Dallas, Dallas, USA

My distinguished colleagues from the University of Tennessee in Memphis have performed an innovative set of complex studies seeking to elucidate somatic mutation variants in patients with MS; across the various clinical subtypes, and compared to normal subjects. Quite surprising to this reviewer, the authors identified a TPNO-1 binding domain change of hnRNPA1-M9; an RNA binding protein that is among the first to be shuttled into and out of the nucleus. Perhaps even more conspicuous is that these somatic modifications are directly adjacent to well characterized mutations that are associated with the development of ALS (i.e. accelerated motor neuron disease of the upper and lower motor neuron compartments).  

My colleagues further show that 8/12 SNVs are shown to occur in primary progressive MS (a slowly and insidiously progressive loss of the upper motor neuron compartment of the CNS. They further elucidated six novel SNVs; with substitutions within the MS IgG epitope of M9 (AA 293-304).

The authors proceed to provide a SNV rate stratification across normal subjects vs clinical subtypes of MS. Here they found the following corresponding rates:

PPMS- 2.21%

SPMS- 1.69%

RRMS- 0.56%

Normal human subjects = 0%

Much has been achieved with the application of genome wide association studies (GWAS), such as the identification of a linkage between the IL-2 and IL-7 receptor genes; immune regulatory elements that dovetail nicely with the higher risk of MS developing in subjects who are also positive for the MHC Class II HLADR21501B1 (2 copies > 1 copy with respect to relative risk). Alternately, MHC Class I HLA-A*02 confers a protective influence. Indeed, it is intriguing that most of the SNPs are associated with CD4+T cell regulation. 

Contemporary screening techniques have further evolved with even greater refinement in genetic element identification conferring risk of MS by virtue of the utilization of the powerful technique of whole exome sequencing (WES), that provides important perspective on differential gene expression mechanisms. 

Finally, the GWAS discovery associated with MS of the CYP27B1 gene, has particular relevance to an epigenetic disorder such as MS, given that this gene encodes for the critical enzyme that serves to transform the 25-OH-D form to the 1,25-OH-D active form of vitamin D. 

With respect to the pathophysiologic relevance of the hnRNPA1 variants, these appear to alter neuronal function, and ultimately culminate in neurodegeneration, in conjunction with serving a role in the important regulation of viral synthesis (another highly conspicuous epigenetic factor with special relevance to MS and related disorders). As the authors rightly point out, to date the somatic mutations in question and under investigation have only been elucidated from PBMCs of MS patients. 

This work, if confirmed, may have special and critical relevance to the highly enigmatic phenomenology of what determine the transition from a relapsing to a progressive form of MS (or a progressive form from the inception without an antecedent relapsing and adaptive immune phase of the disorder), and such studies might shed light on the pathobiological underpinnings of axonal and neuronal degeneration; the histopathological signatures of what we currently believe to constitute the signature of irreversible (at least so far) neurologic disability with corresponding loss of functional capabilities. 

This work, contributes to our deeper understanding of the potential mechanisms of disease that figure prominently in influencing progression and neurodegeneration for MS in particular, but may also shed light on those mechanisms that underly neurodegenerative disorders in general. 

The authors are to be congratulated for this fine contribution to the literature.

I have read this submission. I believe that I have an appropriate level of expertise to confirm that it is of an acceptable scientific standard.

# Response

**Published:** 2014-09-11

**Authors:** Michael Levin

**Affiliations:** University of Tennessee Health Science Center, Memphis, USA

We appreciate Professor Frohman’s insightful commentary about our work. We concur that to date, we have only found somatic nucleotide variants (SNVs) in PBMCs of MS patients. Further studies are warranted about the contribution of SNVs to axonal and neuronal degeneration in the CNS of MS patients. This is addressed in the last two paragraphs of the discussion.

# Peer review: Referee response for version 1

**DOI:** 10.5256/f1000research.4747.r5207

**Published:** 2014-07-23

**Authors:** Michael K. Racke

**Affiliations:** 1 Department of Neurology and Multiple Sclerosis Center, The Ohio State University, Columbus, OH, USA

This manuscript by Lee and Levin continues work by Levin on the role of an immune response to hnRNP-A1 in human demyelinating disorders, including multiple sclerosis. Previously, Levin had shown that an antibody response to HTLV-I Tax cross-reacted with hnRNP-A1. Now, they go on to show that there are mutations in hnRNP-A1 that affect the localization of this protein and how it could affect neuronal survival in multiple sclerosis.In general, the experiments appear to be well performed. One might be interested to know whether testing in another cell line such as a oligodendroglial cell line would have similar effects on cell survival as in the neuroblastoma cell line. This could also be highly relevant in MS, as in addition to neuronal loss, there is demyelination and loss of oligodendrocytes.This work attempts to address whether mutations in hnRNP-A1 could contribute to MS pathogenesis. It would be interesting to note whether antibody responses to the protein correlated with mutations (i.e. does the mutation in the protein affect tolerance). Another issue that needs to be addressed is whether the mutations in the hnRNP-A1 gene are due to increased mutational frequency that can be observed in replicating cells, similar to the observation of increased hprt mutations made by Allegretta many years ago in MBP-specific T cells.Overall, this is an interesting study which certainly increases the interest in non-myelin targets in diseases such as MS.

I have read this submission. I believe that I have an appropriate level of expertise to confirm that it is of an acceptable scientific standard.

# Response

**Published:** 2014-09-11

**Authors:** Michael Levin

**Affiliations:** University of Tennessee Health Science Center, Memphis, USA

We appreciate Professor Racke’s critique, including that the studies were “well performed.” The following concerns expressed by Professor Racke were addressed:

1. **One might be interested to know whether testing in another cell line such as a oligodendroglial cell line would have similar effects on cell survival as in the neuroblastoma cell line. This could also be highly relevant in MS, as in addition to neuronal loss, there is demyelination and loss of oligodendrocytes.**
  We used SK-N-SH neurons as a model system to examine the effect that mutant forms of hnRNP A1 might have on target cell function. Considering that cells other than neurons are clearly involved in the pathogenesis of MS, we plan to add oligodendrocyte cell lines to future studies of mutant hnRNP A1. We addressed this in the sixth paragraph of the discussion.
** **
2. **This work attempts to address whether mutations in hnRNP-A1 could contribute to MS pathogenesis. It would be interesting to note whether antibody responses to the protein correlated with mutations (i.e. does the mutation in the protein affect tolerance).**
  We agree that this would be in important and insightful experiment, one that we think is beyond the scope of the current study. Our previous work showed that MS patients develop antibodies to hnRNP A1 using Western blots. We have not yet developed the technology that would allow us to quantitate the amount of antibodies to hnRNP A1 in individual patients. We will consider this in the future as we examine the potential connection between antibodies to and somatic mutations within hnRNP A1.
3. ****Another issue that needs to be addressed is whether the mutations in the hnRNP-A1 gene are due to increased mutational frequency that can be observed in replicating cells, similar to the observation of increased hprt mutations made by Allegretta many years ago in MBP-specific T cells.****
  We appreciate this insight offered by Professor Racke, which is very relevant to our work and have included it in paragraph 4 of the discussion.

# Peer review: Referee response for version 1

**DOI:** 10.5256/f1000research.4747.r5210

**Published:** 2014-07-03

**Authors:** Hans Lassmann

**Affiliations:** 1 Department Neuroimmunology, Medical University of Vienna, Wien, A-1090, Austria

This is a potentially interesting study, reporting increased presence of novel somatic single nucleotide variants (SNVs) within the RNA binding protein hnRNP A1 in peripheral blood mononuclear cells (PBMCs) in patients with multiple sclerosis (MS) in comparison to controls. The disease associated SNVs resulted in a mis-localization of hnRNP A1 into cytoplasmic stress granules and this mis-localization was associated with cellular apoptosis in cells, transfected with the respective mutants.  From a technical point of view the study is well performed. The question, however, remains what the findings mean for the pathogenesis of multiple sclerosis.It is interesting to see that there is apparently a high incidence of induced SNVs in PBMCs of MS patients, which most likely not only affects the gene investigated in this study. This may be due to the fact that MS is a chronic inflammatory disease, leading to proliferation of leukocytes (in particular T-lymphocytes) in the peripheral immune system. In addition increased oxidative stress in PBMCs of MS patients has been reported before. Both conditions may lead to increased somatic mutation in these cell populations. Thus, in a study like this the inclusion of a control population with long-lasting chronic inflammation would be important in addition to the study of normal controls. According to the presented data, the particular SNVs are also likely to result in cell elimination. Whether this changes the immune response in MS patients has not been investigated in this study.Finally the authors discuss that their findings may have implications for the understanding the mechanisms of tissue injury and neurodegeneration in MS.  To support this conclusion, evidence has to be provided that similar somatic mutations are also present in cells of the central nervous system, such as neurons or oligodendrocytes.  The basic observation of an increased incidence of somatic mutations in PBMCs in MS presented in this article is a first (but preliminary) observation, which is interesting.However, it is not clear what this means for the disease. The key message from the authors that this is an important finding for MS pathogenesis is not substantiated by the data. I agree with the authors that further experiments (possibly beyond the scope of a new version of this article) are needed. For example:

1. To look for somatic mutations for other genes, to see whether the paper just shows that in PBMCs of MS patients there is an increased rate of somatic mutations in many different genes or whether the mutations in the specific gene are MS specific.
2. To expand the sample of control patients, in particular by including patients with other chronic inflammatory diseases (e.g. patients with chronic rheumatoid arthritis).
3. To search for similar mutations in neurons and glia of the MS brain to establish a link to neurodegeneration in this disease.

I have read this submission. I believe that I have an appropriate level of expertise to confirm that it is of an acceptable scientific standard, however I have significant reservations, as outlined above.

# Response

**Published:** 2014-09-11

**Authors:** Michael Levin

**Affiliations:** University of Tennessee Health Science Center, Memphis, USA

We appreciate Professor Lassmann’s comments including that the study was “well performed” and the acknowledgement that “increased incidence of somatic mutations in PBMCs in MS presented in this article is a first…”. We agree with his constructive critiques, which we have addressed below.

1. **To look for somatic mutations for other genes, to see whether the paper just shows that in PBMCs of MS patients there is an increased rate of somatic mutations in many different genes or whether the mutations in the specific gene are MS specific.**
  Our ongoing studies are designed to evaluate more genes. Using next-generation sequencing, which is beyond the scope of this paper, we may be able to assess a broader number of genes, potentially including coding exomes of the human genome. In this study, we evaluated three different regions of hnRNP A1: the C-terminal of PrLD, M9 and hnRNP A1’s C-terminus. Within this subset of genes, we saw a segregation of SNVs. For example, there were similar numbers of SNVs in hnRNP A1’s C-terminus in all groups examined (Table 1, Supplement 2).  In contrast, in PPMS SNVs segregated to the TPNO-1 binding domain of M9 and SPMS to the MS IgG epitope of M9. Although more genes are required to assess their role in MS compared to other diseases, in this study, we have already observed a differential expression of SNVs between types of MS and HCs. This is addressed in the last paragraph of the discussion.
2. **To expand the sample of control patients, in particular by including patients with other chronic inflammatory diseases (e.g. patients with chronic rheumatoid arthritis).**
  We will add patients with other chronic inflammatory diseases such as rheumatoid arthritis to our future studies. Fortunately, there is a large rheumatoid arthritis research group at our institution, which allow access to these samples. We addressed this in the last paragraph of the discussion.
3. **To search for similar mutations in neurons and glia of the MS brain to establish a link to neurodegeneration in this disease.**
  Our ongoing studies will apply the techniques used in this manuscript in central nervous system tissues isolated from MS patients at autopsy. This is addressed in paragraph 6 of the discussion. As we complete these studies, as well as studies examining the role of SNVs on the immune response in MS patients, this will address Professor Lassmann’s concern about these findings being relevant to the pathogenesis of MS.
4. **Professor Lassmann also suggested that oxidative stress might be a cause of SNVs in the PBMCs of MS patients. **
  We added this insight with references to the fourth paragraph of the discussion: “MS is also characterized by increased oxidative stress (in PBMC and brain), which can cause DNA damage and somatic mutations.”
